# Development of a Virtual Human for Supporting Tobacco Cessation During the COVID-19 Pandemic

**DOI:** 10.2196/42310

**Published:** 2023-12-05

**Authors:** Kate Loveys, Erica Lloyd, Mark Sagar, Elizabeth Broadbent

**Affiliations:** 1 Department of Psychological Medicine The University of Auckland Auckland New Zealand; 2 Soul Machines Auckland New Zealand; 3 Auckland Bioengineering Institute The University of Auckland Auckland New Zealand

**Keywords:** virtual human, conversational agent, tobacco cessation, eHealth, COVID-19, public health, virtual health worker, smoking cessation, artificial intelligence, AI, chatbot, digital health intervention, web-based health, mobile phone

## Abstract

People who consume tobacco are at greater risk of developing severe COVID-19. Unfortunately, the COVID-19 pandemic reduced the accessibility of tobacco cessation services as a result of necessary social restrictions. Innovations were urgently needed to support tobacco cessation during the pandemic. Virtual humans are artificially intelligent computer agents with a realistic, humanlike appearance. Virtual humans could be a scalable and engaging way to deliver tobacco cessation information and support. Florence, a virtual human health worker, was developed in collaboration with the World Health Organization to remotely support people toward tobacco cessation during the COVID-19 pandemic. Florence delivers evidence-based information, assists with making quit plans, and directs people to World Health Organization–recommended cessation services in their country. In this viewpoint, we describe the process of developing Florence. The development was influenced by a formative evaluation of data from 115 early users of Florence from 49 countries. In general, Florence was positively perceived; however, changes were requested to aspects of her design and content. In addition, areas for new content were identified (eg, for nonsmoker support persons). Virtual health workers could expand the reach of evidence-based tobacco cessation information and personalized support. However, as they are a new innovation in tobacco cessation, their efficacy, feasibility, and acceptability in this application needs to be evaluated, including in diverse populations.

## Introduction

### Background

Efforts to encourage tobacco cessation have become ever more urgent during the COVID-19 pandemic. Past and present users of tobacco are at greater risk of severe disease, hospitalization to intensive care units, and death when infected by SARS-CoV-2 [1,2]. Tobacco use is a risk factor for health conditions such as cancer, respiratory disease, cardiovascular disease, and diabetes [3,4], which increase the risk of developing severe illness when infected by SARS-CoV-2.

Despite the public health importance of tobacco cessation during the COVID-19 pandemic, there is a severe shortage of support services and resources. According to the World Health Organization (WHO), of the 780 million people worldwide who want to quit tobacco, only 30% have access to the support services that they need to do so successfully [5]. The low accessibility of support services for tobacco cessation has been attributed to a shortage of trained health care workers [6] and the lack of a national tobacco control program in many low-income countries [7]. The COVID-19 pandemic further reduced the accessibility of cessation support as many countries rolled back face-to-face services to prevent disease spread [8]. To address this care gap, the WHO partnered with the private sector to develop digital tools and resources to help people make a successful quit attempt during the year-long World No Tobacco Day “Commit to Quit” campaign [5].

### Digital Interventions for Tobacco Cessation

Digital interventions have been posited to revolutionize how people receive support for tobacco cessation due to their ability to overcome many of the barriers to in-person care (eg, cost and physical restrictions) [7]. Digital interventions may improve access to evidence-based cessation care for low-resource and hard-to-reach populations [7]. Digital interventions for tobacco cessation have included websites that deliver behavioral interventions, educational modules, or tailored videos (with and without professional follow-up); emails containing tailored advice; quitlines; SMS text message services; smartphone apps; and a face-aging simulation to visualize the effects of tobacco use [9,10]. A Cochrane systematic review found that digital interventions that are interactive and tailored to the user resulted in higher quit rates compared to self-help or nonactive controls, including at 6-months follow-up and beyond [9]. However, digital interventions that involve adjunct professional support are typically less feasible in resource-limited settings, and other solutions are needed.

### Virtual Humans

Virtual humans are sophisticated embodied conversational agents with realistic, humanlike appearances that use artificial intelligence to inform their social responses [11]. They are autonomous animations of people presented through a computer, smartphone, or tablet screen and accessed via an internet connection. Further, because of their humanlike form, virtual humans can deliver nonverbal cues for enhanced social and emotional engagement. This may increase engagement with the health information or intervention that they are delivering [12]. Early studies have found that virtual humans can be an effective method for delivering interventions for stress, mental health, diet, exercise, social skills, and improving health literacy [11]. Moreover, virtual humans have been found to be feasible, acceptable, and engaging for delivering a behavioral intervention during the COVID-19 pandemic [13].

Some studies have explored the use of virtual humans as part of tobacco cessation support. The research field is developmental and consists of predominantly pilot and feasibility studies; however, promising results have been found. A pilot randomized controlled trial compared the effect of a virtual human–led tobacco cessation program (“Flexiquit”) that incorporates acceptance and commitment therapy techniques for over 6 sessions to a waitlist control on tobacco consumption in young adults [14]. The virtual human–led program significantly improved perceived self-efficacy to quit tobacco, intention to quit, nicotine dependence, and the average number of cigarettes smoked compared to the control. There was a nonsignificant trend toward the intervention increasing tobacco quit rates relative to the control. Although these results are promising, it is unclear how the intervention would compare to an active control group.

Another study found that a tablet-based virtual human was an acceptable and potentially effective way to administer a tobacco cessation program to military veterans from the United States [15]. The virtual human administered a 14-day program including content from the US clinical practice guidelines for tobacco cessation. The program covered tobacco triggers, motivations and strategies for quitting, relapse prevention strategies, and developing a quit plan. Only 6 participants took part in that study, and most (5/6, 83%) reduced the average number of cigarettes smoked during the program and adopted stricter tobacco smoking rules at home. However, only half of the participants reported trying to quit tobacco smoking. The virtual human facilitator was found to be helpful and user-friendly. All participants used the program daily, followed the virtual human’s advice, felt “very satisfied or satisfied” with the advice, and would recommend the virtual human program to a friend. Another study found that a virtual human was highly acceptable as a screening tool for tobacco use disorder in an outpatient setting [16].

These early pilot trials show promising results for virtual humans as part of individual care for tobacco consumption. However, virtual humans have not been used for tobacco cessation in public health settings across multiple countries. To address this gap, the WHO commissioned the development of Florence, a virtual health worker for supporting tobacco cessation on a global public health website. Florence’s goals were to (1) provide evidence-based health information, (2) assist with making quit plans, and (3) direct people to WHO-recommended cessation services in their country. This viewpoint describes the development of Florence.

### Development of Florence

**Figure 1:**
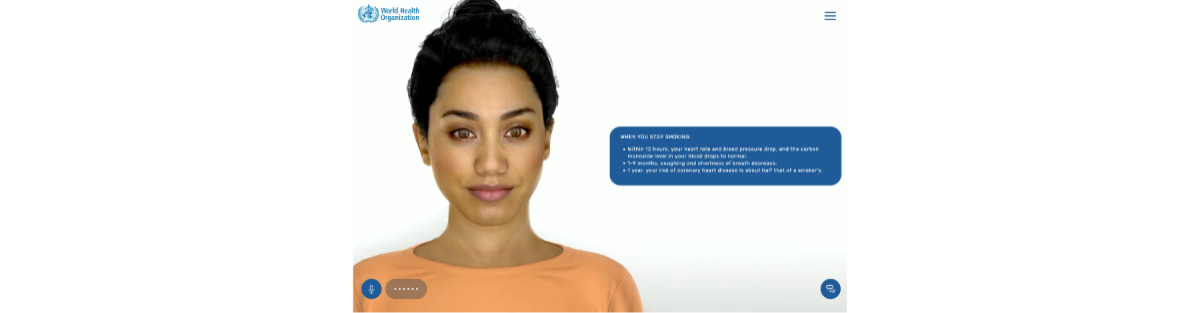
Florence, the World Health Organization’s first virtual health worker (Soul Machines).

Florence, the WHO’s first virtual health worker, was developed by Soul Machines, in collaboration with the WHO, Rooftop, Amazon Web Services, and Google Cloud (see Figure 1). She is available for support 24/7 on the WHO’s publicly accessible website [17]. Florence was launched in September 2020. At the time of the research, Florence could speak English, Spanish, French, Chinese, Arabic, and Russian live on the website.

### Digital Human Technology

Soul Machines Digital Humans are a type of virtual human, which have realistic, humanlike appearances and capabilities driven by artificial intelligence. Soul Machines’ Digital Humans are rendered using advanced computer-generated imagery techniques adapted from Hollywood film makers [18]. They incorporate a sophisticated cognitive architecture with a virtual nervous system powered by artificial neural networks that drive the learning, social, and emotional responses of the Digital Human [18,19]. Soul Machines’ Digital Humans are autonomously animated in real time, responding to the visual and verbal input of their users from webcam data. Emotional information from the user affects the Digital Human’s emotional state and responses. In addition, it is possible to preprogram different personalities to affect their response tendencies (eg, empathetic and extroverted). Florence incorporates Soul Machines’ Digital Human technology to empathetically deliver health information and support to users.

Florence is modeled on a multiracial young adult female. She uses a synthetic female voice to talk with users in a choice of accents consistent with the language she is speaking. Users can choose to communicate with Florence through speech or text in a messenger window to the right of her interface, which enables a more private interaction (especially if headphones are used). Florence uses a finite-state conversation engine with predetermined language content to ensure that the delivery of the intervention materials and health information is accurate and appropriate to the audience and application. She shows pictures and shares links in her interface for an engaging educational experience.

### Intervention Content

#### Information

Florence delivers tobacco cessation content developed by the WHO. This includes evidence-based information on how tobacco use affects health outcomes (eg, increased risk of morbidity and mortality), why quitting is a good idea (eg, immediate health benefits, improved longevity, and financial benefits), and how nicotine replacement therapy can help (eg, what it is, how it is delivered, and its rationale and effectiveness). Florence also provides information about the COVID-19 pandemic, which covers why it is important to quit tobacco during the pandemic, vaccine safety, whether to become vaccinated, what to do after vaccination, types of COVID-19 vaccines, and COVID-19 myths. In addition, users can find information about Florence including what a Digital Human is, who she was developed by, and what her purpose is.

#### Brief Tobacco Cessation Advice

Florence supports users to develop a quit plan in accordance with WHO-recommended brief tobacco intervention strategies and models [20]. Florence assesses how regularly the user consumes tobacco products and their readiness to quit tobacco; helps them to plan a quit date; and provides advice for adhering to the quit plan such as designating support people, telling as many people as possible about the quit plan, and removing all tobacco related products from their environment. Moreover, Florence provides users with the telephone number of their local quitline for professional counseling support and directs people to other WHO-recommended web-based resources for tobacco cessation. These include messenger-based chatbots, the QuitTobacco (WHO) smartphone app, and the quitting toolkit website that links to a range of cessation resources [21].

### Formative Evaluation

A formative evaluation process was used to inform the development of Florence. In total, 115 users completed an anonymous web-based survey in which they rated different aspects of the virtual health worker and provided qualitative feedback. It was a naturalistic survey open to all users who engaged with the English version of Florence between April 2021 and April 2022. No other eligibility criteria were set. The option to participate was presented to users at the end of their interaction experience. Users were neither targeted nor was a sample size set. Data were not collected on how respondents found Florence’s web page on the WHO website. However, it is possible that they were exploring the tobacco cessation section of the WHO’s website, they may have Google searched for tobacco cessation support, or they may have seen the WHO’s press release on Florence.

**Figure 2:**
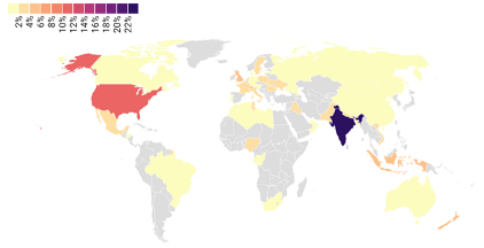
Geographic spread of survey respondents.

Data were collected between April 19, 2021, and April 5, 2022, from users across 49 unique countries (Figure 2). All survey respondents were included in the analyses, including those who reported that they were a nonsmoker in their qualitative data. This decision was made because it allowed a fuller picture of who was using Florence to inform future design and content decisions. Florence was hosted on the WHO’s website and was freely available in all countries in 6 languages at the time of the survey. Only the results of a survey for the English version of Florence are presented due to feasibility constraints of posting the survey in other languages.

Respondents were from an approximately equal spread of high-income (49/115; 42.6%) and lower-middle income countries (50/115; 43.5%). Fewer respondents were from upper-middle income (16/115; 13.9%) and low-income countries (0/115; 0%). Most respondents were from India where English is 1 of 2 national languages (25/115; 21.7%) and the United States (10/115; 8.7%). Respondent countries were determined from IP addresses, and country income levels were derived from the World Bank country classifications by income level for 2022 to 2023 [22]. No demographic data were collected on the respondents.

Users were presented with a link to complete an optional survey at the end of their interaction with Florence. Survey respondents were users who had anonymously accessed Florence’s website of their own accord; they were not recruited or approached to visit the website. No demographic or identifiable data were collected. The survey asked about perceptions of Florence and her advice through the following questions: *“*How was your overall experience with Florence as a virtual persona?” and “How do you rate the information and advice given by Florence?” Users responded on a 4-point scale with response options (1=poor, 2=needs improvement, 3=good, and 4=excellent). The survey also asked users about their behavioral intent to take initial steps to quit tobacco after their interaction with Florence (“Has your interaction with Florence helped you to make a quit plan?” and “Do you plan to try any other cessation services recommended by Florence?”). Respondents used a 4-point scale with response options (1=no, 2=probably not, 3=maybe, and 4=yes). Lastly, users were asked, “Do you have any other feedback?” to which they were able to provide open-ended qualitative feedback on the virtual health worker. The items were designed by the researchers to evaluate Florence’s performance at achieving her goals of providing quality information and advice and motivating and supporting people to quit tobacco. Data were analyzed using SPSS statistics software (version 28; IBM Corp). Missing data were handled using pairwise deletion. Quantitative data were analyzed by computing the mean scores or frequencies of responses, depending on the measure. Qualitative data were analyzed by 1 researcher (KL) using reflexive thematic analysis methods in accordance with Braun and Clarke’s [23] guidelines.

On average, users reported a good overall experience with Florence as a virtual persona (n=114; mean 3.17, SD 0.82 out of 4). Users reported that Florence provided good information and advice (n=114; mean 3.21, SD 0.92 out of 4). The majority (50/114; 43.8%) of users reported that their interaction with Florence helped them to make a quit plan. Some users (37/114; 32.5%) reported that Florence may have helped. Few users reported that Florence did not help with making a quit plan (15/114; 13.2%) or probably did not help (12/114; 10.5%). Most users (50/112; 44.6%) reported they planned to try the tobacco cessation services that Florence had recommended to them. Almost as many users (49/112; 43.8%) reported that they would maybe try the services recommended by Florence. Very few users were not willing to try the services recommended by Florence (4/112; 3.6%) or would probably not try the services (9/112; 8%). The data suggested to the development team that generally, Florence’s design and content was positively received by users. However, some changes could be made to how Florence initiates developing a quit plan and to her design.

To provide further insight on the ratings, qualitative feedback was received from 73 (63.5%) out of 115 users. The themes from the qualitative feedback are presented in Table 1. In summary, users identified aspects of the virtual health worker that they liked (eg, informative, sense of connection, and clear communication) and that could be improved (eg, more personalization and interactivity, and a more humanlike voice). Some participants expressed enthusiasm for the virtual human experience, whereas 1 participant expressed a preference for a human interaction. In addition, through the qualitative feedback, 7 (6.1%) out of 114 respondents identified themselves as nonsmokers.

**Table 1:** Parent themes and subthemes from user feedback on Florence. Some participants had more than 1 theme in their responses.

Parent theme, subtheme, and subsubtheme				Participants, n^a^		Representative quote
**Virtual human intervention strengths**				9		—^b^
	Informative		4		“I didn’t need to quit because I don’t smoke, but the information was very helpful.” [ID093]	
	Sense of connection		1		“I didn’t need to quit because I don’t smoke, but the information was very helpful.” [ID093]	
	Clear communication		1		“Clear, strong, and fairly informative.” [ID044]	
	**Useful**		3		—	
		—	2		“Very useful, thanks!” [ID061]	
		Suited to the COVID-19 pandemic	1		“It was a great plan during this covid situation.” [ID091]	
**Virtual human intervention suggested improvements**				32		—
	**Virtual human interaction**		23		—	
		More open-ended conversation	2		“I don’t like that you can only ask fixed questions.” [ID002]	
		Speech-to-text accuracy	4		“Difficulties [from Florence] about understanding my english [which could be bettered, I’m French. Or my mic disfunctions maybe].” [ID029]	
		Slower pace	1		“Felt rather rushed.” [ID017]	
		Reinforce advice	2		“…strong reinforcement of this advice by florence is not present. It can be improved in that aspect.” [ID091]	
		Repeat options	1		“Doesn’t repeat options, rather goes back right to the beginning of the interaction.” [ID027]	
		Personalization	2		“It’s not personalised.” [ID044]	
		More conversation topics	2		“Needs regular conversation on topics not directly associated with smoking. I think, it would be good to have at least virtual communication facing such emotional problems- as relatives are not always available.” [ID089]	
		More interactivity	2		“It seems a little too structured rather than interactive.” [ID081]	
		Lip sync and facial movements	3		“Sync between the mouth movement and voice can be better.” [ID026]	
		More humanlike voice	1		“The bot still sounds very robotic.” [ID027]	
		Show intelligence	2		“Is it possible to make her more smart?” [ID059]	
		Memory	1		“It would be cool if florence can remember what information she has given me and doesn’t give it to me again. Almost like a tick box of information shared?” [ID113]	
		More languages	1		“Need Chinese version.” [ID114]	
	**Tobacco cessation program**		8		—	
		A more detailed quit plan	2		“The interaction should be more detailed and quit plan must be made by date to date.” [ID006]	
		A more efficient quit plan	1		“The plan seems not very efficient. They are just some ideas.” [ID074]	
		Content for nonsmokers	4		“Maybe make the questions relevant for people who do not smoke.” [ID086]	
		Include evidence on the effectiveness of AI^c^	1		“I am very curious about the effectiveness of florence. Is there any research to provide evidence about the effectiveness of AI when quitting smoking?” [ID034]	
	**Interface**		2		—	
		Option to link to human support	1		“Chat box might be helpful where there are real people.” [ID032]	
		Android app	1		“Please make as an android application of this as an assistant that will help much more.” [ID065]	
Enthusiasm for the virtual human experience				15		“I love it.” [ID068]
Preference for a human interaction				1		“Not a substitute for human interaction for me.” [ID046]
No feedback				65		“None.” [ID115]

^a^The number of participants who talked about the parent theme, subtheme, or subsubtheme in their data.

^b^Not available.

^c^AI: artificial intelligence.

## Discussion

### Principal Findings

Florence, the WHO’s first virtual health worker, delivers evidence-based information about tobacco use and guides people toward making a quit plan. Florence was the first virtual human to be deployed in a global public health application across multiple languages. A formative evaluation influenced the development process and revealed that, on average, Florence was rated as providing a good user experience and good health information and advice. Most participants reported that Florence helped them to make a quit plan and that they intended to visit the cessation resources she recommended. Areas for improvement were identified pertaining to Florence’s design and content, which are discussed below. Moving forward, trials are needed to examine Florence’s efficacy, feasibility, and acceptability for tobacco cessation support in diverse populations.

### Lessons Learned and Future Directions

The user evaluations revealed areas for improvement to direct development. First, it became evident that information was needed for nontobacco users and support persons looking to help someone quit, as not all users of Florence were tobacco consumers. Changes were also necessary to aspects of Florence’s design. In particular, Florence’s conversation design needed to be more open-ended, interactive, and personalized and cover a broader range of topics. Indeed, highly interactive and tailored digital interventions have been shown to be more effective for tobacco cessation compared to nonactive controls in a recent systematic review [9]. Some users felt that Florence could adopt strategies to help them understand the content, for example, delivering the information at a slower pace, reinforcing the advice, and repeating options. Similar strategies were requested for a virtual quit coach in another study and may be helpful for improving comprehension of health information [15]. Some users wanted more variety in the languages that Florence could speak. Since the feedback, Hindi has been added to Florence’s language set. Changes to Florence’s lip sync, vocal realism, memory, and language understanding were also requested, and these features are being updated as the underlying technology advances.

Florence’s content on tobacco cessation was generally rated positively; however, some users would have liked the quit plan to be more detailed with a more efficient, succinct set of steps. In addition, some users would have liked to access Florence from a smartphone app for convenience (note that users could access Florence from a smartphone web browser at the time of the survey) or to have had the option to connect with a human over a chat window in places. These suggestions have been echoed in prior research with a virtual quit coach, suggesting that they may be important features for virtual human tobacco cessation programs more broadly [15].

Moving forward, it will be important to implement strategies for promoting longer-term engagement with Florence. Florence could expand her capabilities to deliver a more comprehensive cessation program (eg, covering more topics, with more personalized information and advice based on user data). Florence should also regularly update her content (eg, to suit global events such as the COVID-19 pandemic) and engage in more rapport-building behaviors for relational engagement (eg, self-disclosure and empathy) [24-26]. Moreover, Florence’s appearance should be culturally tailored based on user data or choice to boost acceptability. It is unclear whether Florence is best used as an educational resource on the WHO website, as a resource on the websites of local public health or primary care services, as a personal smartphone app, or as all of these. Florence’s visibility should be increased through enhanced search engine optimization techniques and increased promotion so that she can easily be found by people wanting to quit tobacco.

### Limitations and Future Research and Development

This viewpoint describes the development of a novel public health tool, a virtual health worker. The development process, including the formative evaluation, had several limitations that could be addressed in future work.

First, qualitative data from the formative evaluation revealed that users were a mix of tobacco users, support persons, and educators. The presence of nonsmokers in the sample may have affected responses in the user survey. These user groups are likely to vary in their technology perceptions and requirements. Subsequent formative evaluations of Florence could deliberately recruit targeted user groups (eg, tobacco users and support persons) to inform the development of the content most relevant to each group. Our formative evaluation of all users enabled us to observe that nonsmoking support persons and educators also visit Florence. This suggested to the development team that Florence should include information relevant to those user groups and to tailor her language accordingly.

Second, demographic data were not collected; thus, it is difficult to assess the generalizability of the feedback. Follow-up on research and development efforts should consult users from different age groups, genders, and ethnicities as well as across low-middle and high-income countries to inform the design and content. Although our survey respondents were from 49 unique countries, many countries had only 1 or 2 respondents; thus, the group sizes were too small to compare responses by country. Moreover, it is possible that users in countries where English was not a national language had higher education and socioeconomic status relative to others residing in those countries. Future research and development work could recruit larger samples from specific countries to enable comparison. To understand the cultural acceptability of Florence, future research could compare the data of users interacting with Florence in different languages.

Moreover, as the user survey was voluntary, it is possible that there could have been a sample bias that affected the results, with respondents who felt especially positive or negative about the technology. However, our results did not appear to skew to the extreme ends of our rating scales as might be expected with this form of response bias.

This paper describes the development of a prototype technology and does not include an evaluation of acceptability. To date, it is unclear how other tobacco cessation technologies (eg, a text-only website) might compare to Florence on these outcomes. However, based on prior research, we would expect the interactive virtual health worker to perform more highly [9]. Future research should examine how the virtual human compares to other forms of tobacco cessation support (eg, a text-only website, a helpline, and in-person advice from a health care professional).

Lastly, the public health impact of this intervention is yet to be examined and evaluations are needed to see if Florence is associated with uptake of cessation treatment (eg, nicotine replacement therapy and medications) and reductions in tobacco consumption. To aid in the evaluation, baseline data should be collected on the willingness of participants to quit smoking and their prior experience with receiving tobacco cessation advice and making quit attempts.

The strengths of this viewpoint are that it describes the development of a novel virtual health worker that can be used as part of a global public health program. To inform the development of the virtual health worker, insights were derived from a global user survey conducted in an ecologically valid, naturalistic setting. Future research should evaluate the effect of virtual health workers in tobacco cessation and other public health programs, given they are a highly scalable and socially engaging manner through which health information and support could be provided. Moreover, virtual health workers could facilitate public health interventions in novel interaction environments including augmented and virtual reality and the metaverse.

### Conclusions

Virtual health workers have the potential to improve the accessibility of tobacco cessation information and support during a pandemic. This viewpoint described the development of Florence, the WHO’s first virtual health worker that provides information and encourages quit attempts. The development process was influenced by a formative evaluation of preliminary user data. Overall, Florence was viewed positively by users; however, areas for improvement were identified. These included changes to Florence’s tobacco cessation program (eg, a more detailed and efficient quit plan and content for support persons), the virtual human design (eg, more personalization, a slower pace, and more conversation topics), and the interface (eg, a smartphone app and the option to speak to a human). Research is needed to investigate her acceptability and effectiveness at reducing tobacco use as part of a public health program.

## Abbreviations

WHO: World Health Organization
